# Community Comfort With Automatic Sharing of Race, Ethnicity, and Language Data Between Health Care Settings: Cross-Sectional Study

**DOI:** 10.2196/67288

**Published:** 2025-10-06

**Authors:** Noah Brazer, Baylah Tessier-Sherman, Deron Galusha, Sakinah C Suttiratana, Corrine Liu, Katherine K Kim, Mark E Abraham, Marcella Nunez-Smith, Karen H Wang

**Affiliations:** 1 Department of Internal Medicine, Yale School of Medicine, 100 Church Street South, Suite A200, New Haven, CT, 06519, United States, +1 (203) 785-5233; 2 Department of Public Health Sciences, School of Medicine, University of California Davis, Sacramento, CA, United States; 3 DataHaven, New Haven, CT, United States

**Keywords:** health care disparities, health care justice, data sharing, data autonomy, race, ethnicity, disparity, race, ethnicity, and language, racial identity, ethnic identity, REL

## Abstract

**Background:**

Little is known regarding patient attitudes toward automatic sharing of race, ethnicity, and language (REL) data in health care settings despite the universal practice of data sharing across health care institutions and providers.

**Objective:**

This study aims to assess public comfort with disclosing and automatically sharing REL data in health care settings and understand the social factors associated with these attitudes.

**Methods:**

Using the 2022 DataHaven Community Wellbeing Survey from 1196 adult Connecticut residents, we examined factors associated with public comfort with disclosing and automatically sharing REL data across health care settings. We generated unadjusted and adjusted logistic models to examine associations between factors and responses to the data-sharing questions.

**Results:**

Most residents surveyed were White (n=873, 73%), followed by African American or Black (n=167, 14%), Asian or Native Hawaiian or other Pacific Islander (n=31, 2.6%), multiracial (n=31, 2.6%), and American Indian or Alaska Native (n=12, 1%). The majority of respondents were not Hispanic or Latino (n=1051, 87.9%). More than half of respondents reported excellent or very good self-rated health (SRH; n=635, 53.1%), and most participants reported almost always trusting their health care provider (n=939, 78.5%). Most participants reported being willing to share race and ethnicity data at a hospital or clinic (n=1008, 84.3%) and REL data automatically (n=947, 79.2%) in health care settings. Hispanic or Latino (adjusted odds ratio [AOR] 0.049, 95% CI 0.25-0.94) and multiracial (AOR 0.32, 95% CI 0.14-0.76) respondents were less likely to be willing to disclose race and ethnicity data compared to those who were not Hispanic or Latino and who were White, respectively. Individuals who sometimes trust health care providers (AOR 0.57, 95% CI 0.35-0.94) or rarely/never (AOR 0.35, 95% CI 0.15-0.85) were less likely to be willing to disclose race and ethnicity data than those who almost always trust health care providers. African American or Black (AOR 0.46, 95% CI 0.29-0.72) and American Indian or Alaska Native (AOR 0.18, 95% CI 0.04-0.75) individuals were less likely to be willing to share REL data automatically than White individuals. Those who sometimes trust health care providers (AOR 0.48, 95% CI 0.31-0.74) or rarely/never trust health care providers (AOR 0.25, 95% CI 0.11-0.56) were less likely to be willing to share REL data automatically than those who almost always trust health care providers. Those with poor/fair SRH versus very good/excellent SRH were less likely to be willing to share REL data automatically (AOR 0.54, 95% CI 0.34-0.85).

**Conclusions:**

Racial and ethnic identity, SRH, and trust in health care providers affect willingness to share REL information with providers and other health systems.

## Introduction

Patient data collection pertaining to race, ethnicity, and language (REL) was first mandated in the United States as part of the Civil Rights Act of 1964 as a means to document race-based discrimination in health care settings and drive institutional and social change [1]. This groundbreaking legislative change was one of the first steps to improve health care disparities among racially and ethnically minoritized people who face marginalization because of systemic oppression. In recent years, REL data collection has become digitized following the passing of the Health Information and Technology for Economic and Clinical Health Act (HITECH) in 2009, which used monetary incentives to encourage the use of electronic health record (EHR) systems [2]. Despite being codified as a federal mandate, REL data collection for patients is not always implemented systematically in health care settings, nor is it always accurate [3]. For example, during the initial height of the COVID-19 pandemic, race and ethnicity data were missing from as many as 56% of reported cases at certain points in time for various public health surveillance data [4]. As a result, health care and public health systems and state government agencies have invested in ensuring improved REL data collection in health [5].

Though race and ethnicity data collection in health care systems is the main mechanism to examine quality of care across diverse populations based on racialized identities, there have been long-standing challenges to consistent data collection despite various improvement efforts [6]. These challenges include concern among clinical staff about asking patients about their information and a lack of training for clinical staff on the importance of data collection. Furthermore, if patients do not have a clear understanding of why these data are being collected and whether the data will be used outside of a clinical setting for research purposes, they may feel less comfortable disclosing information. Efforts to overcome these obstacles to data collection have included reassuring patients about the confidentiality of their data, as well as introducing scripts and training for clinical staff. Beyond individual system-level quality improvement efforts, health systems have been using and sharing this information with other systems across health care settings, which can help standardize data collection practices [7]. While data sharing across health systems and organizations serves as both a mechanism to support public health and streamline care for patients, it may occur without patients’ knowledge or explicit consent [1,8-10]. Several prior studies have specifically examined patients’ preferences regarding sharing of health data with other clinical providers, finding that patients prefer to be asked permission before having their data shared electronically outside of emergency contexts and that patients who are unwilling to share their data automatically with other health care providers cited concerns about potential breaches in security [11-13]. Although these studies focused on the automatic sharing of general health data, little is known about how patients in the United States feel about sharing specific aspects of their medical records, such as REL data, that are embedded in their EHR [14,15]. One study found no significant difference in patients’ willingness to share general health data automatically based on their race and ethnicity; however, this study did not specifically investigate attitudes toward automatic sharing of REL data [16].

This study aims to understand how adults aged 18 and up in Connecticut feel about having their REL data collected and automatically shared with health care systems and whether there are differences in their preferences associated with their racial and ethnic identities, as well as other sociodemographic factors.

## Methods

### Study Design

This cross-sectional study involved analysis of data from the 2022 DataHaven Community Wellbeing Survey [17]. The DataHaven Community Wellbeing Survey is conducted on an ongoing basis in Connecticut and is designed to measure well-being and quality of life at the statewide, town, and zip code level. The cost of fielding the survey is shared across more than 100 public and private agencies, including all of the state’s acute care hospitals, large community foundations, and major cities, as well as many agencies representing rural and suburban communities.

### Setting

Survey data were collected by the Siena College Research Institute between August 1 and August 28, 2022. Respondents to the 2022 DataHaven Community Wellbeing Survey were contacted via cell phone and landline or were part of an online exchange. The landline sample included both listed and unlisted telephone numbers, using random digit dialing. The cell phone sample was drawn from dedicated wireless telephone exchanges within Connecticut. Dynata’s Wireless LITe database was utilized, which enabled targeting of cell phone samples by region or zip code. The online sample was provided by Lucid, a market research platform that runs an online exchange for survey respondents. The samples drawn from this exchange matched a set of demographic quotas on age, gender, and region. Respondents were sent from Lucid directly to the survey software operated by the Siena College Research Institute. All respondents taking the survey online completed an attention check before and during the survey to ensure proper attention was being paid throughout. The cell phone and landline interviews were conducted in English or Spanish, while online surveys were conducted in English. All respondents were screened for residence in the state of Connecticut.

### Participants

The survey sample included 1196 respondents randomly selected from Connecticut residents, aged 18 years or older, to inform state-level estimations of resident well-being, equity, and quality of life, as previously described [17]. The only inclusion criterion for the survey was being a Connecticut resident aged 18 years or older. Among the respondents, 727 (60.8%) completed the survey on a cell phone, 321 (26.8%) completed it on a landline, and 148 (12.4%) completed it online. Respondents represented all 169 Connecticut towns. We followed the STROBE (Strengthening the Reporting of Observational Studies in Epidemiology) guidelines for a cross-sectional study for reporting [18].

### Assessments and Data Sources

The outcome variables for this study were delevoped from 2 questions that were added to the 2022 DataHaven Community Wellbeing Survey. The first question was “Patients are often asked their race and ethnicity at a hospital or clinic. Are you OK sharing this information at a hospital or clinic?” Response options were “Yes” or “No.” The second question was “If you were offered the choice to have your race/ethnicity/language information automatically shared electronically with the different places where you receive medical care, how likely would you be to agree to it?” This question was adapted from a prior study [11]. Response options fell along a 4-point Likert scale from “Very likely” to “Very unlikely.” “Very likely” and “Somewhat likely” were collapsed into one category, while “Somewhat unlikely” and “Very unlikely” were collapsed into one category.

The covariates for this study were race and ethnicity, gender, age, primary language, reporting of chronic disease, self-rated health (SRH), trust in health care provider, and prior experience with discrimination in health care settings. We selected these covariates as prior research suggests they may influence the relationship between a person and their comfort with sharing personal data for either research or health care use [12,19-21]. Race categories were “African American or Black,” “American Indian or Alaska Native,” “Asian,” “Native Hawaiian or Other Pacific Islander,” “White,” and “other/something else.” Ethnicity categories were “Hispanic or Latino” and “not Hispanic or Latino.” Asian and Native Hawaiian or other Pacific Islander were collapsed into one category because of small sample size. Respondents who selected more than one race category were placed in the “Multiracial” category. Chronic disease information was collected by asking respondents about the following conditions: high blood pressure or hypertension; diabetes; heart attack, also called myocardial infarction; angina or coronary heart disease; stroke; and asthma. Respondents who indicated they had any of the conditions were grouped into one category. Respondents with “Excellent” or “Very good” SRH were grouped into one category, while those with “Fair” or “Poor” SRH were grouped into one category. Trust in health care providers was collected by asking how often the following statement was true: “I trust that my health care provider is trying to do what is best for me.” Response options were “Almost always,” “Sometimes,” “Rarely,” and “Never.” “Rarely” and “Never” were collapsed into one category. Prior experience of discrimination in health care settings was collected by asking, “When seeking health care, have you ever been treated with less respect or received services that were not as good as what other people get?”

### Study Size

For our analytic approach, we first conducted univariate analysis to describe survey participants who had any data available for analysis (n=1196). We then removed participants who were missing outcome data (n=69) or covariate data (n=134), leaving a final analytic sample of 993.

### Data Analysis

We conducted unadjusted and adjusted parsimonious logistic models to compare demographic characteristics and responses to the data-sharing questions and to determine the independent predictors of our outcomes. We entered covariates with a *P* value <.20 from the unadjusted models into the multivariable regression models using backward elimination, retaining covariates with a *P* value <.05. For the multivariate adjusted model analyzing comfort with REL disclosure, the following variables remained in the parsimonious model: race, ethnicity, gender, and trust in health care provider. For the multivariate adjusted model analyzing comfort with REL sharing, the following variables remained in the parsimonious model: race, age, trust in health care provider, self-rated overall health, and any chronic diseases. Correlations between covariates were examined, and no significant collinearity was observed. All analyses were conducted using SAS Version 9.4 (SAS Institute).

### Ethical Considerations

This study did not meet the regulatory criteria for human subjects research because the data from the 2022 DataHaven Community Wellbeing Survey comprise a publicly accessible deidentified database, and as such, institutional review board review and approval were not required [22]. During data collection, informed consent was obtained by DataHaven, and identifiable data was removed before the data were made publicly accessible.

## Results

Among the sample of 1196 adult Connecticut residents aged 18 years or older who were randomly selected to participate in the 2022 DataHaven Community Wellbeing Survey, half of the respondents identified as female (Table 1). The majority of residents surveyed were White (n=873, 73%), followed by African American or Black (n=167, 14%), Asian or Native Hawaiian or other Pacific Islander (n=31, 2.6%), multiracial (n=31, 2.6%), and American Indian or Alaska Native (n=12, 1%). Most respondents were not Hispanic or Latino (n=1051, 87.9%). This population slightly overrepresented African American or Black, Native Hawaiian or other Pacific Islander, and American Indian or Alaska Native groups while underrepresenting White, Asian, and Hispanic or Latino groups relative to the Connecticut population reported in the most recent census [23]. In the cohort, 22.1% (n=264) of individuals were between the ages of 18 and 39 years. Only a small percentage of the participants did not speak English as their primary language (n=64, 5.4%). The majority of respondents reported excellent or very good SRH (n=635, 53.1%), and more than half reported a chronic disease condition (n=668, 55.9%). Most participants reported almost always trusting their health care provider (n=939, 78.5%), and the majority did not have a prior experience of discrimination in a health care setting (n=990, 82.8%). Most participants were willing to share race and ethnicity data at a hospital or clinic (n=1008, 84.3%) and were willing to share REL data automatically (n=947, 79.2%; Table 1).

**Table 1.** Survey participant characteristics and responses (N=1196).

	Participants, n (%)	
Race		
African American/Black	167 (13.9)	
American Indian/Alaska Native	12 (1.0)	
Asian/Native Hawaiian or other Pacific Islander	31 (2.6)	
White	873 (72.9)	
Multiracial	31 (2.6)	
Missing	82 (6.9)	
Ethnicity		
Hispanic or Latino	126 (10.5)	
Not Hispanic or Latino	1051 (87.9)	
Missing	19 (1.6)	
Age (years)		
18‐39	264 (22.1)	
40‐64	428 (35.8)	
65+	455 (38)	
Missing	49 (4.1)	
Gender		
Female	602 (50.3)	
Male	591 (49.4)	
Other	3 (0.2)	
English as primary language?		
Yes	1122 (93.8)	
No	64 (5.4)	
Missing	10 (0.8)	
Trust health care provider		
Almost always	939 (78.5)	
Sometimes	194 (16.2)	
Rarely/never	42 (3.5)	
Missing	21 (1.8)	
Self-rated overall health		
Excellent/very good	635 (53.1)	
Good	338 (28.3)	
Fair/poor	219 (18.3)	
Missing	4 (0.3)	
Any chronic disease reported?		
Yes	668 (55.8)	
No	519 (43.4)	
Missing	9 (0.8)	
Prior experience with discrimination		
Yes	170 (14.2)	
No	990 (82.8)	
Don’t know	29 (2.4)	
Missing	7 (0.6)	
Okay sharing race/ethnicity?		
Yes	1008 (84.3)	
No	142 (11.9)	
Missing	46 (3.8)	
Likelihood to automatically share race, ethnicity, and language data		
Very likely/somewhat likely	947 (79.2)	
Somewhat unlikely/very unlikely	216 (18.1)	
Missing	33 (2.8)	

Unadjusted models suggested that the likelihood of being willing to share race and ethnicity data at a hospital or clinic was associated with a respondent’s race, ethnicity, gender, and primary language and how much they trust their health care provider (Table 2). In the multivariate logistic regression model, multiracial participants were significantly less likely to be willing to share their race/ethnicity data compared to White participants (adjusted odds ratio [AOR] 0.32, 95% CI 0.14-0.76; Table 2). Similarly, Hispanic or Latino participants were significantly less likely to be willing to share their race/ethnicity data compared to those who were not Hispanic or Latino (AOR 0.49, 95% CI 0.25-0.94). Female respondents were more likely to be willing to share their race/ethnicity data compared to male respondents (AOR 1.59, 95% CI 1.07-2.41). Individuals who reported they only sometimes (AOR 0.57, 95% CI 0.35-0.94) or rarely/never (AOR 0.35, 95% CI 0.15-0.85) trust that their health care provider has their best interest in mind were less likely to be willing to share race/ethnicity data compared to those who almost always trust their health care provider.

**Table 2.** Association between individual characteristics and comfort with disclosing race and ethnicity at hospitals or clinics.

Demographic characteristics	Unadjusted		Multivariate (adjusted)	
	Odds ratio (95% CI)	*P* value	Odds ratio (95% CI)	*P* value
Race				
White	Reference	—^a^	Reference	—
African American/Black	0.70 (0.41-1.20)	.19	0.68 (0.39-1.17)	.16
American Indian/Alaska Native	0.37 (0.08-1.79)	.21	0.57 (0.11-3.02)	.51
Asian/Native Hawaiian or other Pacific Islander	0.80 (0.24-2.73)	.72	1.20 (0.33-4.29)	.78
Multiracial	0.29 (0.12-0.67)	.004^b^	0.32 (0.14-0.76)	.01^c^
Ethnicity				
Not Hispanic or Latino	Reference	—	Reference	—
Hispanic or Latino	0.44 (0.24-0.83)	.01^c^	0.49 (0.25-0.94)	.03^c^
Age (years)				
18‐39	Reference	—	—	—
40‐64	0.84 (0.49-1.45)	.53	—	—
65+	0.96 (0.56-1.66)	.89	—	—
Gender				
Male	Reference	—	Reference	—
Female	1.55 (1.03-2.34)	.04^c^	1.59 (1.07-2.41)	.03^c^
English as primary language				
Yes	Reference	—	—	—
No	0.41 (0.18-0.93)	.03^c^	—	—
Trust health care provider				
Almost always	Reference	—	—	—
Sometimes	0.53 (0.32-0.86)	.01^c^	0.57 (0.35-0.94)	.03^c^
Rarely/never	0.27 (0.12-0.64)	.003^b^	0.35 (0.15-0.85)	.02^c^
Self-rated overall health				
Excellent/very good	Reference	—	—	—
Good	0.87 (0.55-1.37)	.54	—	—
Fair/poor	0.99 (0.57-1.74)	.98	—	—
Any chronic diseases reported?				
No	Reference	—	—	—
Yes	0.83 (0.55-1.26)	.39	—	—
Prior experience with discrimination in health care?				
No	Reference	—	—	—
Yes	1.24 (0.66-2.33)	.51	—	—

a Not applicable.

b *P*<.01.

c *P*<.05.

In unadjusted models, an individual’s likelihood of being willing to share REL data automatically with various health care locations was associated with their race, SRH, and how much they trust their health care provider (Table 3). In the multivariate logistic regression model, African American or Black (AOR 0.46, 95% CI 0.29-0.72) and American Indian or Alaska Native respondents (AOR 0.18, 95% CI 0.04-0.75) were significantly less likely to be willing to share REL data automatically compared to White respondents (Table 3). Respondents who were aged 40‐64 years (AOR 0.56, 95% CI 0.34-0.92) and 65+ years (AOR 0.51, 95% CI 0.30-0.87) were significantly less likely to be willing to share REL data than those aged 18‐39 years. Individuals who reported they only sometimes (AOR 0.48, 95% CI 0.31-0.74) or rarely/never (AOR 0.25, 95% CI 0.11-0.56) trust that their health care provider has their best interest in mind were also significantly less likely to be willing to share REL data automatically than those who almost always trust their health care provider. Respondents with fair/poor SRH were also significantly less likely to be willing to share REL data automatically compared to those with very good/excellent SRH (AOR 0.54, 95% CI 0.34-0.85). In contrast, respondents who reported having any chronic disease were significantly more likely to be willing to share REL data than those who reported not having any chronic disease (AOR 1.58, 95% CI 1.10-2.31).

**Table 3.** Association between individual characteristics and willingness to automatically share race, ethnicity, and language data with different health care locations.

Demographic characteristics	Unadjusted		Multivariate (adjusted)	
	Odds ratio (95% CI)	*P* value	Odds ratio (95% CI)	*P* value
Race				
White	Reference	—^a^	Reference	—
African American/Black	0.48 (0.31-0.73)	<.001^b^	0.46 (0.29-0.72)	<.001^b^
American Indian/Alaska Native	0.20 (0.05-0.77)	.02^c^	0.18 (0.04-0.75)	.02^c^
Asian/Native Hawaiian or other Pacific Islander	0.68 (0.25-1.84)	.45	0.57 (0.20-1.65)	.30
Multiracial	0.38 (0.17-0.85)	.02^c^	0.44 (0.19-1.02)	.56
Ethnicity				
Not Hispanic or Latino	Reference	—	—	—
Hispanic or Latino	0.86 (0.46-1.61)	.63	—	—
Age (years)				
18‐39	Reference	—	Reference	—
40‐64	0.70 (0.44-1.11)	.13	0.56 (0.34-0.92)	.02^c^
65+	0.87 (0.54-1.38)	.54	0.51 (0.30-0.87)	.01^c^
Gender				
Male	Reference	—	—	—
Female	0.92 (0.66-1.28)	.62	—	—
English as primary language				
Yes	Reference	—	—	—
No	1.28 (0.49-3.33)	.62	—	—
Trust health care provider				
Almost always	Reference	—	—	—
Sometimes	0.49 (0.32-0.74)	<.001^b^	0.48 (0.31-0.74)	<.001^b^
Rarely/never	0.21 (0.10-0.45)	<.001^b^	0.25 (0.11-0.56)	<.001^b^
Self-rated overall health				
Excellent/very good	Reference	—	Reference	—
Good	1.21 (0.79-1.84)	.39	1.18 (0.76-1.84)	.46
Fair/poor	0.54 (0.36-0.81)	.003^d^	0.54 (0.34-0.85)	.008^d^
Any chronic diseases reported?				
No	Reference	—	Reference	—
Yes	1.24 (0.88-1.73)	.22	1.58 (1.10-2.31)	.02^c^
Prior experience with discrimination in health care?				
No	Reference	—	—	—
Yes	0.67(0.43-1.05)	.08	—	—

a Not applicable.

b *P*<.001.

c *P*<.05.

d *P*<.01.

Sensitivity analyses showed consistent adjusted results using 2 additional race/ethnicity categorization methods: the common method of grouping race/ethnicity prior to the new 2024 United States Office of Management and Budget race/ethnicity collection guidelines (Tables S1 and S2 in Multimedia Appendix 1) and the rarest classification method to enumerate small populations (Tables S3 and S4 in Multimedia Appendix 1) [24].

## Discussion

### Principal Results

In general, the majority of Connecticut respondents are comfortable disclosing their race and ethnicity and having their REL data shared automatically. However, racially and ethnically minoritized individuals are less comfortable disclosing race and ethnicity information at hospitals and clinics and having their REL data automatically shared with health institutions where they receive care. As compared to respondents who were White and were not Hispanic or Latino, multiracial and Hispanic or Latino respondents were the least likely to be willing to disclose their race and ethnicity data, respectively, while Black or African American and American Indian or Alaska Native respondents were less likely to be willing to have their REL data automatically shared between medical providers.

### Comparison With Prior Work

While previous studies showed no significant difference in respondents’ willingness to automatically share general health data between race and ethnicity identity groups [11,16], the results of this study add a more nuanced view of specific data elements within the EHR that certain minoritized individuals feel less inclined to share automatically. It is possible that prior studies saw no significant difference in willingness to automatically share general health data because respondents were not explicitly informed about all the sensitive data that comprised their EHR, such as REL data. Additionally, the previous studies were conducted in California, which has a substantially different representation of race and ethnicity both in the survey samples and the state population than in the current study in Connecticut, with higher representation of Hispanic or Latino and Asian or Native Hawaiian or other Pacific Islander respondents and lower representation of Black or African American respondents. In the United States, Black, Indigenous, and Latino communities have faced discrimination when accessing health care systems, leading to poorer health outcomes and health care delivery than their White counterparts [25,26]. These discriminatory experiences may contribute broadly to diminished comfort with REL data collection and automatic sharing [27].

The finding that lower SRH was independently associated with a decreased likelihood of being willing to share REL data automatically warrants further examination. Past studies have demonstrated a relationship between race and ethnicity and SRH [28-30]. Furthermore, studies investigating the relationship between SRH and likelihood to share data with physicians have shown that those with lower SRH are more likely to share information with their physician, which is consistent with our finding that individuals with chronic disease were more likely to be willing to share REL data [31,32]. However, among our study population, no correlation was found between SRH and chronic disease, suggesting that Connecticut residents may use other metrics to determine their SRH beyond chronic disease. Given the findings of this study, it may be possible that individuals with lower SRH are more comfortable with sharing general health data specifically for treatment purposes but draw the line at sharing REL data, whereas those with chronic disease are comfortable sharing all elements of their data. Follow-up studies could further explore relationships between one’s social identities, health status, and sharing of information.

Our finding that trust in health care providers is highly associated with comfort with sharing is consistent with past studies that have established a link between patient trust in their provider and willingness to disclose or share health information [19,33]. Additionally, a previous study found differences in health information seeking behavior of individuals by race and ethnicity, with Black and Hispanic individuals, compared to White individuals, more trusting of health information from media, charitable organizations, and religious organizations and Hispanic individuals, compared to White individuals, less trusting of and willing to seek information from doctors [34]. The results of our study further highlight the importance of trust between patient and provider and reveal that individuals with decreased levels of trust in their health care providers may be hesitant to disclose and share any data, whether health-specific or demographic. Our initial work in this area suggests that trust in health care providers may mediate these relationships between the correlates of interest and outcomes (data not shown). Future studies can explore these relationships.

### Limitations

This study had several limitations. Namely, the number of individuals identifying as American Indian or Alaska Native or Native Hawaiian or other Pacific Islander was very low, which affected the statistical analyses. Given that individuals who identify as American Indian or Alaska Native or Native Hawaiian or other Pacific Islander tend to be minoritized in most areas of the United States, a larger cohort would be needed to better understand the nuances of comfort with REL data disclosure and sharing among these communities. Additionally, the 2022 DataHaven Community Wellbeing Survey was only conducted in English or Spanish, which limits representation of communities in Connecticut that do not speak either of these two languages. Further, the population of Connecticut is not representative of that of the United States, and therefore, generalizations made while using these results should be done with caution.

### Conclusion

The results of this study have implications for how we operationalize health information exchange across clinical and public health settings in the current discussion of how to most effectively enhance the nationwide health data ecosystem and standardize social and structural drivers of health data [35]. This study suggests that nuances exist in how people think about race and ethnicity data and the sharing of specific information within their health record. People’s individual backgrounds likely influence their comfort with sharing sensitive data and their comfort with that data being shared automatically with other health settings. More work is needed to gauge whether patients have an understanding of what components comprise their health record, which of these components may be shared, and with whom the data are being shared.

## Supplementary material

10.2196/67288 Multimedia Appendix 1 Sensitivity analyses comparing two additional race/ethnicity categorization methods.

## Abbreviations

AOR: adjusted odds ratio
EHR: electronic health record
HITECH: Health Information and Technology for Economic and Clinical Health Act
REL: race, ethnicity, and language
SRH: self-rated health
STROBE: Strengthening the Reporting of Observational Studies in Epidemiology
