# Supplementary material for: Community Comfort With Automatic Sharing of Race, Ethnicity, and Language Data Between Health Care Settings: Cross-Sectional Study
Source: Interact J Med Res. 2025 Oct 6;14:e67288. doi: 10.2196/67288 (PMC12500222; doi:10.2196/67288)
Supplement: Multimedia Appendix 1 [file ijmr-v14-e67288-s001.docx]

Table S1: Association between Individual Characteristics and Comfort with Disclosing Race and Ethnicity at Hospitals or Clinics (Common Race/Ethnicity Groupings)

| Demographic Characteristic | Unadjusted | | Multivariate (adjusted) | |
| --- | --- | --- | --- | --- |
|  | OR (95% CI) | p-value | OR (95% CI) | p-value |
| Race/Ethnicity |  |  |  |  |
| White | Reference | | Reference | |
| African American/Black | 0.69 (0.39,1.22) | 0.20 | 0.68 (0.38,1.21) | 0.19 |
| American Indian/Alaska Native | 0.39 (0.04,3.53) | 0.40 | 0.44 (0.05,4.03) | 0.47 |
| Asian/Native Hawaiian or Other Pacific Islander | 0.83 (0.19,3.66) | 0.80 | 0.96 (0.21,4.33) | 0.96 |
| Hispanic/Latino | 0.33 (0.20,0.55) | <0.001*** | 0.36 (0.21,0.61) | <0.001*** |
| Multiracial | 0.26 (0.11,0.60) | 0.002** | 0.29 (0.12,0.68) | 0.005** |
| Age |  |  |  |  |
| 18-39 years | Reference | | N/A | |
| 40-64 years | 0.93 (0.55,1.54) | 0.77 |  |  |
| 65+ years | 0.94 (0.57,1.56) | 0.81 |  |  |
| Gender |  |  |  |  |
| Male | Reference | | Reference | |
| Female | 1.60 (1.08,2.36) | 0.018* | 1.60 (1.07,2.38) | 0.022* |
| Trust healthcare provider |  |  |  |  |
| Almost always | Reference | |  | |
| Sometimes | 0.52 (0.33,0.83) | 0.006** | 0.57 (0.36,0.92) | 0.022* |
| Rarely/Never | 0.33 (0.14,0.75) | 0.008** | 0.42 (0.18,0.98) | 0.044* |
| Self-rated overall health |  |  |  |  |
| Excellent/Very good | Reference | | N/A | |
| Good | 0.88 (0.56,1.38) | 0.59 |  |  |
| Fair/Poor | 0.77 (0.47,1.27) | 0.31 |  |  |
| English as primary language |  |  |  |  |
| Yes | Reference | | N/A | |
| No | 0.33 (0.18,0.62) | <0.001*** |  |  |
| Any chronic diseases reported? |  |  |  |  |
| No | Reference | | N/A | |
| Yes | 0.78 (0.53,1.16) | 0.22 |  |  |
| Prior experience with discrimination in healthcare? |  |  |  |  |
| No | Reference | | N/A | |
| Yes | 1.28 (0.70,2.34) | 0.4304 |  |  |

^a^ *, p<0.05

^b^ **, p<0.01

^c^ ***, p<0.001

Table S2: Association between Individual Characteristics and Willingness to Automatically Share REL Data with Different Healthcare Locations (Common Race/Ethnicity Groupings)

| Demographic Characteristic | Unadjusted | | Multivariate (adjusted) | |
| --- | --- | --- | --- | --- |
|  | OR (95% CI) | p-value | OR (95% CI) | p-value |
| Race/Ethnicity |  |  |  |  |
| White | Reference | | Reference | |
| African American/Black | 0.50 (0.32,0.78) | 0.002** | 0.47 (0.30,0.75) | 0.002** |
| American Indian/Alaska Native | 0.11 (0.02,0.66) | 0.016* | 0.08 (0.01,0.55) | 0.010** |
| Asian/Native Hawaiian or Other Pacific Islander | 0.61 (0.20,1.88) | 0.39 | 0.46 (0.14,1.48) | 0.19 |
| Hispanic/Latino | 0.69 (0.41,1.16) | 0.17 | 0.69 (0.39,1.22) | 0.20 |
| Multiracial | 0.43 (0.19,1.00) | 0.049* | 0.47 (0.19,1.13) | 0.090 |
| Age |  |  |  |  |
| 18-39 years | Reference | | Reference | |
| 40-64 years | 0.70 (0.45,1.09) | 0.12 | 0.47 (0.28,0.80) | 0.005** |
| 65+ years | 0.80 (0.51,1.26) | 0.34 | 0.28 (0.13,0.60) | 0.001** |
| Gender |  |  |  |  |
| Male | Reference | | N/A | |
| Female | 0.95 (0.69,1.32) | 0.76 |  |  |
| Trust healthcare provider |  |  |  |  |
| Almost always | Reference | |  | |
| Sometimes | 0.49 (0.33,0.74) | <0.001*** | 0.49 (0.32,0.74) | <0.001*** |
| Rarely/Never | 0.24 (0.12,0.50) | <0.001*** | 0.28 (0.13,0.60) | 0.001** |
| Self-rated overall health |  |  |  |  |
| Excellent/Very good | Reference | | Reference | |
| Good | 1.20 (0.79,1.81) | 0.39 | 1.18 (0.76,1.81) | 0.46 |
| Fair/Poor | 0.52 (0.35,0.77) | 0.001** | 0.52 (0.33,0.81) | 0.004** |
| English as primary language |  |  |  |  |
| Yes | Reference | | N/A | |
| No | 0.94 (0.47,1.90) | 0.87 |  |  |
| Any chronic diseases reported? |  |  |  |  |
| No | Reference | | Reference | |
| Yes | 1.20 (0.86,1.67) | 0.28 | 1.58 (1.09,2.30) | 0.017* |
| Prior experience with discrimination in healthcare? |  |  |  |  |
| No | Reference | | N/A | |
| Yes | 0.68 (0.44,1.06) | 0.092 |  |  |

^a^ *, p<0.05

^b^ **, p<0.01

^c^ ***, p<0.001

Table S3: Association between Individual Characteristics and Comfort with Disclosing Race and Ethnicity at Hospitals or Clinics (Rarest Race/Ethnicity Groupings)

| Demographic Characteristic | Unadjusted | | Multivariate (adjusted) | |
| --- | --- | --- | --- | --- |
|  | OR (95% CI) | p-value | OR (95% CI) | p-value |
| Race/Ethnicity |  |  |  |  |
| White | Reference | | Reference | |
| African American/Black | 0.62 (0.36,1.04) | 0.071 | 0.61 (0.36,1.04) | 0.067 |
| American Indian/Alaska Native | 0.33 (0.12,0.93) | 0.035* | 0.40 (0.14,1.15) | 0.089 |
| Asian/Native Hawaiian or Other Pacific Islander | 0.47 (0.19,1.17) | 0.11 | 0.56 (0.22,1.43) | 0.23 |
| Hispanic/Latino | 0.32 (0.18,0.56) | <0.001*** | 0.34 (0.20,0.60) | <0.001*** |
| Age |  |  |  |  |
| 18-39 years | Reference | | N/A | |
| 40-64 years | 0.93 (0.55,1.54) | 0.77 |  |  |
| 65+ years | 0.94 (0.57,1.56) | 0.81 |  |  |
| Gender |  |  |  |  |
| Male | Reference | | Reference | |
| Female | 1.60 (1.08,2.36) | 0.018* | 1.59 (1.07,2.38) | 0.022* |
| Trust healthcare provider |  |  |  |  |
| Almost always | Reference | |  | |
| Sometimes | 0.52 (0.33,0.83) | 0.006** | 0.57 (0.35,0.91) | 0.019* |
| Rarely/Never | 0.33 (0.14,0.75) | 0.008** | 0.41 (0.17,0.96) | 0.040* |
| Self-rated overall health |  |  |  |  |
| Excellent/Very good | Reference | | N/A | |
| Good | 0.88 (0.56,1.38) | 0.59 |  |  |
| Fair/Poor | 0.77 (0.47,1.27) | 0.31 |  |  |
| English as primary language |  |  |  |  |
| Yes | Reference | | N/A | |
| No | 0.33 (0.18,0.62) | <0.001*** |  |  |
| Any chronic diseases reported? |  |  |  |  |
| No | Reference | | N/A | |
| Yes | 0.78 (0.53,1.16) | 0.22 |  |  |
| Prior experience with discrimination in healthcare? |  |  |  |  |
| No | Reference | | N/A | |
| Yes | 1.28 (0.70,2.34) | 0.4304 |  |  |

^a^ *, p<0.05

^b^ **, p<0.01

^c^ ***, p<0.001

Table S4: Association between Individual Characteristics and Willingness to Automatically Share REL Data with Different Healthcare Locations (Rarest Race/Ethnicity Groupings)

| Demographic Characteristic | Unadjusted | | Multivariate (adjusted) | |
| --- | --- | --- | --- | --- |
|  | OR (95% CI) | p-value | OR (95% CI) | p-value |
| Race/Ethnicity |  |  |  |  |
| White | Reference | | Reference | |
| African American/Black | 0.50 (0.33,0.76) | 0.001** | 0.47 (0.30,0.74) | 0.001** |
| American Indian/Alaska Native | 0.16 (0.07,0.39) | <0.001*** | 0.18 (0.07,0.46) | <0.001*** |
| Asian/Native Hawaiian or Other Pacific Islander | 0.79 (0.32,1.95) | 0.61 | 0.67 (0.26,1.76) | 0.42 |
| Hispanic/Latino | 0.82 (0.45,1.48) | 0.50 | 0.82 (0.44,1.55) | 0.54 |
| Age |  |  |  |  |
| 18-39 years | Reference | | Reference | |
| 40-64 years | 0.70 (0.45,1.09) | 0.12 | 0.60 (0.37,0.98) | 0.039* |
| 65+ years | 0.80 (0.51,1.26) | 0.34 | 0.51 (0.30,0.86) | 0.011* |
| Gender |  |  |  |  |
| Male | Reference | | N/A | |
| Female | 0.95 (0.69,1.32) | 0.76 |  |  |
| Trust healthcare provider |  |  |  |  |
| Almost always | Reference | |  | |
| Sometimes | 0.49 (0.33,0.74) | <0.001*** | 0.49 (0.32,0.75) | 0.001** |
| Rarely/Never | 0.24 (0.12,0.50) | <0.001*** | 0.30 (0.14,0.67) | 0.003** |
| Self-rated overall health |  |  |  |  |
| Excellent/Very good | Reference | | Reference | |
| Good | 1.20 (0.79,1.81) | 0.39 | 1.17 (0.76,1.81) | 0.47 |
| Fair/Poor | 0.52 (0.35,0.77) | 0.001** | 0.52 (0.33,0.81) | 0.004** |
| English as primary language |  |  |  |  |
| Yes | Reference | | N/A | |
| No | 0.94 (0.47,1.90) | 0.87 |  |  |
| Any chronic diseases reported? |  |  |  |  |
| No | Reference | | Reference | |
| Yes | 1.20 (0.86,1.67) | 0.28 | 1.60 (1.10,2.33) | 0.014* |
| Prior experience with discrimination in healthcare? |  |  |  |  |
| No | Reference | | N/A | |
| Yes | 0.68 (0.44,1.06) | 0.092 |  | |

^a^ *, p<0.05

^b^ **, p<0.01

^c^ ***, p<0.001

**Supplemental Methods**

For the common method of categorizing race and ethnicity categories prior to the release of the 2024 OMB guidelines, we grouped together all individuals who selected the “Hispanic or Latino” ethnicity, regardless of their race selection. The “Multiracial” category was used to encompass all individuals who selected the “not Hispanic or Latino” ethnicity and two or more race categories (**Supplemental Table 1; Supplemental Table 2**). For the rarest classification method, individuals who reported more than one race and individuals who selected any race category and Hispanic or Latino ethnicity were placed into the category associated with whichever race or ethnicity category they chose that has the least representation in Connecticut, based on previous research to enumerate small populations.
